# MicroRNA In Lung Cancer: Novel Biomarkers and Potential Tools for Treatment

**DOI:** 10.3390/jcm5030036

**Published:** 2016-03-09

**Authors:** Kentaro Inamura, Yuichi Ishikawa

**Affiliations:** Division of Pathology, The Cancer Institute, Japanese Foundation for Cancer Research, 3-8-31 Ariake, Koto-ku, Tokyo 135-8550, Japan; kentaro.inamura@jfcr.or.jp

**Keywords:** adenocarcinoma, carcinoma, driver mutation, histology, miRNA, molecular pathology, morphology, plasma, mutation, oncology, serum, sputum

## Abstract

Lung cancer is the leading cause of cancer death in men and women worldwide. The lack of specific and sensitive tools for early diagnosis as well as still-inadequate targeted therapies contribute to poor outcomes. MicroRNAs are small non-coding RNAs, which regulate gene expression post-transcriptionally by translational repression or degradation of target mRNAs. A growing body of evidence suggests various roles of microRNAs including development and progression of lung cancer. In lung cancer, several studies have showed that certain microRNA profiles classified lung cancer subtypes, and that specific microRNA expression signatures distinguished between better-prognosis and worse-prognosis lung cancers. Furthermore, microRNAs circulate in body fluids, and therefore may serve as promising biomarkers for early diagnosis of lung cancer as well as for predicting prognosis of patients. In the present review, we briefly summarize microRNAs in the development and progression of lung cancer, focusing on possible applications of microRNAs as novel biomarkers and tools for treatment.

## 1. Introduction

Lung cancer is the leading cause of cancer death in men and women worldwide, accounting for more than 1.5 million deaths per year [1]. Lung carcinoma is generally classified as either small-cell lung carcinoma (SCLC) (about 20% of all lung carcinomas) or non-small cell lung carcinoma (NSCLC) (about 80%). Within these groups, further distinctions are made, with NSCLC sub-divided into adenocarcinoma (about 50% of all lung carcinomas), squamous cell carcinoma (SqCC), and large cell carcinoma.

Although new molecular targeted therapies to some types of lung cancer have shown promising results, no potential targeted therapy can be applied to a large number of lung cancer patients. Despite improvements in early diagnosis of lung cancer, most lung cancers are diagnosed at an advanced stage. Therefore, the identification of novel diagnostic biomarkers or treatment strategies is critical and essential for the control of lung cancer.

MicroRNAs, which are small non-coding RNAs that range in size from 19 to 25 nucleotides, play important regulatory roles in animals and plants by targeting mRNAs for translational repression or degradation. MicroRNAs comprise one of the most abundant classes of gene regulatory molecules in multicellular organisms and likely influence the output of many protein-coding genes [2]. A growing body of evidence is emerging to suggest a wide range of fundamental cellular processes such as cell differentiation, proliferation, growth, mobility, and apoptosis, as well as carcinogenesis or cancer progression [3,4]. The expression patterns of microRNAs are likely to correlate with characteristic clinicopathological parameters in cancer subtypes [5], suggesting that microRNAs are potential biomarkers for different cancer subtypes, classified by origin, histology, aggressiveness, or chemosensivity [6,7].

Public database, miRBase (http://www.mirbase.org/) (last access: 9 February 2016) provides various aspects of microRNA information, and the annotated human microRNAs of 1881 have been registered in miRBase 21. In general, the list of microRNAs involved in lung cancer is not limited to several microRNAs, but much wider, as well as the potential diagnostic microRNAs in tissues.

Of importance, an abundance of undegradated microRNAs exists not only in tissues but also in body fluids, including blood, plasma, serum, and sputum [8]. This nature of easy availability makes microRNAs promising biomarkers for non-invasive liquid biopsy in cancer practice. Biomarkers analyzed by liquid biopsy [9,10] include circulating tumor cells and exosomes (containing DNA, mRNA, microRNA, *etc.*) [11,12] as well as circulating cell-free DNA [13], mRNA [14], and microRNA [15]. Although, non-invasive liquid biopsy is promising, it has not been employed for multiclass cancer diagnostics due to non-specificity of these blood-based biosources to pinpoint the nature of the primary cancer [9,13,14].

In addition, microRNAs are far less degradated in formalin-fixed paraffin-embedded (FFPE) samples than mRNAs. Therefore, accurate measurements of microRNAs can be performed from FFPE samples, which are usually collected and preserved in hospitals. An availability of archived FFPE samples for the accurate measurement of microRNAs is a great advantage to conduct microRNA research or apply microRNA profiles for clinical practice.

Due to the characteristic nature of microRNAs, microRNAs have a potential to be used for the development of diagnostics, prognostics, and targeted therapeutics. In terms of treatment, microRNA expression profiles can predict chemotherapeutic response and serve as important biomarkers for the stratification of patients for personalized therapeutic strategies. Furthermore, microRNAs have the potential to serve as molecular targeted agents. Although the initial results from human-based studies revealed the promise of microRNA targeted therapies, several obstacles need to be overcome prior to the therapeutic application of microRNAs to the clinic [16].

In this review, we summarize recent works of microRNAs in lung cancer, focusing on microRNAs as novel biomarkers and potential tools for treatment.

## 2. MicroRNA Biogenesis

**Figure 1:**
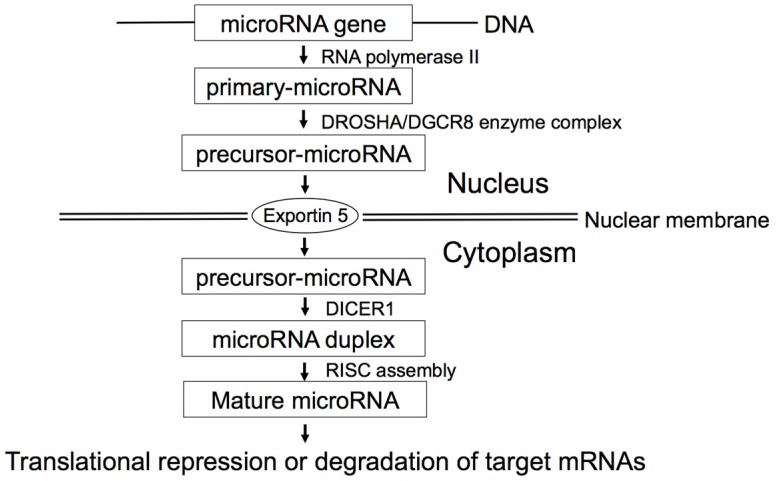
MicroRNA biogenesis. MicroRNAs are initially transcribed by RNA polymerase II as primary-microRNAs with hairpin structures. DROSHA/DGCR8 enzyme complex then cleaves primary-microRNAs into precursor-microRNAs, which are transported to cytoplasm by Exportin 5, and cleaved by DICER to form microRNA duplexes. One strand is selected to function as a mature microRNA and loaded into the RNA-induced silencing complex (RISC), whereas the partner microRNA* is preferentially degraded. The mature microRNA with RISC binds to 3′UTR of target mRNA resulting in translational repression or degradation.

Biogenesis of microRNA (Figure 1) begins with the transcription of primary-microRNA by RNA polymerase II. Then, DROSHA/DGCR8 enzyme complex crops the primary-microRNA into precursor-microRNA, followed by exportin-5-mediated transport from the nucleus to the cytoplasm. Subsequently, DICER1 cleaves the precursor-microRNA to form the microRNA duplex. One strand of the microRNA duplex is selected to function as a mature microRNA and loaded into the RNA-induced silencing complex (RISC), whereas the partner microRNA* strand is preferentially degradated. The mature microRNA leads to translational repression or degradation of target mRNAs.

## 3. MicroRNAs as Tumor Suppressor Genes and Oncogenes

MicroRNAs play important regulatory roles in carcinogenesis not only as tumor suppressor genes but also as oncogenes. Several microRNAs are dysregulated in lung cancer. Table 1 shows principal microRNAs involved in the development or progression of lung cancer, their gene targets, and associated biological processes.

### 3.1. Tumor Suppressor microRNAs

#### 3.1.1. *Let-7* Family

The *let-7* family was the first identified microRNA in humans [17]. In lung cancer, *let-7* has been shown to inhibit the expression of oncogenes involved in cellular proliferation, such as *RAS*, *MYC*, and *HMGA2* [18,19]. *Let-7* also inhibits the expression of *CDK6*, and the reduced expression of *let-7*, therefore, leads to the promotion of cell cycle progression [19]. Of interest, *let-7* directly down-regulates *DICER1* expression, suggesting that *let-7* may regulate the global production of microRNAs [20].

#### 3.1.2. *miR-34* Family

The *miR-34* family (*miR-34a*, *miR-34b*, and *miR-34c*) is directly induced by TP53 in response to DNA damage, controlling cell cycle arrest and apoptosis in cancer [21]. The *miR-34* family is down-regulated in lung cancer, leading to the up-regulation of *miR-34* target genes, such as *MET*, *BCL2*, *PDGFR-α* (*PDGFRA*), and *PDGFR-β* (*PDGFRB*) [22,23,24]. The up-regulation of *MET* and *BCL2* by reduced *miR-34* expression leads to cell proliferation. *MiR-34* dependent PDGFR-α/β downregulation inhibits tumorigenesis and enhances TRAIL (TNF-related apoptosis-inducing ligand)-induced apoptosis in lung cancer [24].

#### 3.1.3. *miR-200* Family

The *miR-200* family (*miR-200a*, *miR-200b*, *miR-200c*, and *miR-429*) plays an important role in the promotion of epithelial-mesenchymal transition (EMT). Through the regulation of ZEB (zinc finger E-box-binding homeobox) transcription factors (*ZEB1* and *ZEB2*), *E-cadherin* (*CDH1*), *vimentin* (*VIM*), the down-regulated *miR-200* family promotes EMT in the progression of lung cancer [25,26].

### 3.2. Oncogenic microRNAs

#### 3.2.1. *miR-21*

*miR-21* is one of the most representative oncogenic microRNAs, being overexpressed in various types of solid tumors as well as leukemia. *miR-21* drives tumorigenesis through inhibition of negative regulators of RAS/MEK/ERK pathway and suppression of apoptosis. Overexpressed *miR-21* downregulates the expressions of *PTEN* [27], *PDCD4* [28,29,30], and *TPM1* [30], promoting cell proliferation and migration, and inhibiting apoptosis.

#### 3.2.2. *miR-17-92* Cluster

The *miR-17-92* polycistronic cluster comprises seven different microRNAs (*miR-17-3p*, *miR-17-5p*, *miR-18a*, *miR-19a*, *miR-20a*, *miR-19b-1*, and *miR-92a*) and resides in intron 3 of the *C13orf25* gene at 13q31.3 [31]. The *miR-17-92* cluster was reported to be overexpressed in lung cancer, in particular SCLC [31]. Overexpression of *miR-17-92* cluster down-regulates *E2F1*, *HIF1A*, and *PTEN*, promoting cell proliferation and cancer progression [32,33].

#### 3.2.3. *miR-221/222*

*miR-221* and *miR-222* are involved in the development and progression of lung cancer by targeting *PTEN* and *TIMP3* tumor suppressor genes [34,35]. Overexpressed *miR-221/222* inhibits apoptosis and promotes cell migration by down-regulating PTEN and TIMP3.

## 4. Diagnostic microRNAs

### 4.1. Diagnostic microRNAs in Tissues

Early detection of lung cancer is prerequisite to reduce lung cancer mortality, because lung cancers are often diagnosed at advanced stages, where clinical treatments are less (or least) effective. MicroRNA expression signatures of lung cancer have been reported by numerous studies, however the reported microRNA profiles were not so consistent. Vosa *et al.* performed a comprehensive meta-analysis of 20 published microRNA expression studies in lung cancer, including a total of 598 tumor and 528 normal lung tissues [36]. Using a recently published robust rank aggregation method, they identified a statistically significant microRNA meta-signature of seven up-regulated (*miR-21*, *miR-210*, *miR-182*, *miR-31*, *miR-200b*, *miR-20*5, and *miR-183*) and eight down-regulated (*miR-126-3p*, *miR-30a*, *miR-30d*, *miR-486-5p*, *miR-451a*, *miR-126-5*p, *miR-143*, and *miR-145*) microRNAs.

### 4.2. Diagnostic microRNAs in Body Fluids

Importantly, microRNAs are present not only in tissues but also in body fluids, such as blood, plasma, serum, or sputum. By examining body fluids, we may be able to distinguish lung cancer patients from healthy individuals. Jiang’s group conducted several studies to assess the usefulness of microRNAs in body fluids for the lung cancer screening [37,38,39,40]. Using sputum specimens, they examined the expression of *miR-21*, which is an overexpressed microRNA in lung cancer [37]. The expression of *miR-21* in sputum was higher in NSCLC patients, and the detection of *miR-21* expression produced 70% sensitivity and 100% specificity in the distinction between 23 NSCLC patients and 17 cancer-free individuals. In another study, using an independent set, they demonstrated that the expression profile of four sputum microRNAs (*miR-21*, *miR-486*, *miR-375*, and *miR-200b*) demonstrated 81% sensitivity and 92% specificity in the distinction between 64 NSCLC patients and 58 healthy individuals [40]. They also assessed the usefulness for plasma microRNAs as potential biomarkers for NSCLC [39]. They showed that the expression profile of four plasma microRNAs (*miR-21*, *miR-126*, *miR-210*, and *miR-486-5p*) yielded 86% sensitivity and 97% specificity in distinguishing 58 NSCLC patients from 29 healthy individuals. Furthermore, this panel of microRNAs produced 73% sensitivity and 97% specificity in identifying stage I NSCLC patients, suggesting the usefulness of plasma microRNAs as potential biomarkers to identify even early stage NSCLC patients [39]. In another study, they showed that the expression profile of three plasma microRNAs (*miR-21*, *miR-210*, and *miR-486-5p*) produced 75% sensitivity and 85% specificity in the distinction between 32 patients with malignant solitary pulmonary nodules (SPNs) and 33 individuals with benign SPNs. SPNs have been increasingly diagnosed with the advancement and widespread use of computed tomography (CT) scan. The combination of microRNA testing and CT scan may serve as a minimally invasive method of diagnosing individuals with SPNs. All of these four studies by Jiang’s group [37,38,39,40] included *miR-21* as a biomarker to distinguish lung cancer patients from healthy individuals. Because *miR-21* is overexpressed in various types of cancer, further studies are warranted to examine *miR-21* expression for the distinction between lung cancer patients and patients of the other cancers.

## 5. MicroRNAs as Biomarkers for Histological Classification

Recent advances in the treatment of NSCLC with new drugs require an appropriate histological subtyping at diagnosis to avoid hazardous side effects. For instance, bevacizumab (brand name: Avastin), a monoclonal antibody which blocks angiogenesis by inhibiting vascular endothelial growth factor A (VEGFA), cannot be used for SqCC patients due to serious hemorrhagic complications. Similarly, pemetrexed (brand name: Alimta), a chemotherapy drug belonging to a class of chemotherapeutic drugs known as folate antimetabolites, cannot be used for SqCC patients due to adverse responses. Several studies have been conducted to distinguish between SqCC and non-SqCC NSCLC, utilizing microRNAs profiles [41,42,43]. Lebanony *et al.* found that higher expression of *miR-205* was specific to SqCC in a test set. In an independent validation set, *miR-205* expression in FFPE samples yielded 96% sensitivity and 90% specificity in the distinction between SqCCs and non-SqCC NSCLCs [41]. As a replication study, Bishop *et al.* showed that the measurement of *miR-205* expression in small biopsies/aspirates can distinguish between SqCCs and non-SqCC NSCLCs [42]. Recently, Hamamoto *et al.* have reported that the expression profile of three microRNAs (*miR-205*, *miR-196b*, and *miR-375*) distinguished between SqCCs and adenocarcinomas with 85% sensitivity and 83% specificity in a validation set [43].

SCLC, one of the neuroendocrine tumors, shows increased expression of *ASCL1*, which is a master gene of neuroendocrine differentiation. Nishikawa *et al.* demonstrated that *miR-357* expression was induced by ASCL1 in lung neuroendocrine carcinoma [44]. They showed that the increased expression of *miR-375* was prerequisite for the neuroendocrine differentiation by ASCL1, and suggested that *miR-375* might reduce the YAP1-related proliferative arrest by inhibiting YAP1.

## 6. Prognostic microRNAs

### 6.1. Investigation for Prognostic microRNAs

As is the case with mRNA, microRNA profiles have been investigated as potential prognostic biomarkers. In 2004, Takamizawa *et al.* focused on microRNA *let-7* [45], and reported that *let-7* expression is lower in lung cancer than in normal lung tissue, and that the lower expression of *let-7* in lung cancer was associated with poor prognosis. In addition, overexpression of *let-7* in A549 lung adenocarcinoma cell line inhibited cell growth. This study [45] is important with respect to the first report of reduced expression of *let-7* and the potential clinical and biological effects of such a microRNA alteration in lung cancer. We examined *let-7* expressions in adenocarcinoma *in situ* (AIS, the new name for BAC (bronchioloalveolar carcinoma) [46]) as well as in invasive adenocarcinoma to investigate the association of *let-7* expression with the progression of lung adenocarcinoma [47]. Of note, even in AIS, the expression of *let-7* was reduced in comparison to matched normal lung tissue, suggesting that *let-7* expression was reduced in the early stage of lung carcinogenesis. AIS is categorized into two subtypes, non-mucinous and mucinous AIS. Interestingly, the expression of *let-7* was lower in mucinous AIS than non-mucinous AIS. The differential expression of *let-7* between two morphological subtypes of AIS suggests an association between microRNA expression and morphology (even in the same category of AIS). According to this observation, complexity of microRNA expressions depending on morphology as well as cancer progression or driver mutations can be presumed.

In 2006, the first microRNA profiling in lung cancer was reported by Yanaihara *et al.* [48]. Using 104 NSCLCs (65 adenocarcinomas and 39 SqCCs) and matched normal lung tissues, they conducted profiling analyses of microRNA expression by microarray. They identified the higher expression of *miR-155* and the lower expression of *let-7a-2* as biomarkers of poor prognosis for NSCLC patients, and confirmed the validity using an independent set by real-time RT-PCR. Subsequently, in 2008, Yu *et al.* [49] identified a five-microRNA signature (*let-7a*, *miR-221*, miR*-137*, *miR-372*, and miR-182*) that classified a training set of 56 NSCLC cases into good-prognosis and poor-prognosis group. Then, they confirmed that the five-microRNA signature divided 62 NSCLC testing cases into two groups with good and poor prognosis. Furthermore, they validated the five-microRNAs signature as a prognostic classifier using an independent cohort set of 62 NSCLCs. This five-microRNA signature worked as a prognostic classifier for NSCLCs even with the same stage or SqCCs as well as adenocarcinomas.

### 6.2. Integrated Prognostic Classifier for Stage I Lung Cancer

*MiR-21* is one of the overexpressed microRNAs in various types of cancer, and targets of *miR-21* include tumor suppressor genes, such as *PTEN* [27,50] and *PDCD4* [28,29,30]. Harris’ group has identified an integrated prognostic classifier for stage I lung adenocarcinoma based on microRNA, mRNA, and DNA methylation biomarkers, using frozen specimens [51]. This integrated prognostic classifier consisted of *miR-21*, four protein-coding genes (*XPO1*, *BRCA1*, *HIF1A*, and *DLC1*), and *HOXA9* promoter methylation. *miR-21*, four protein-coding genes, or *HOXA9* promoter methylation could independently classified stage I adenocarcinomas into two groups with a different survival (hazard ratio (HR) = 2.3, *p* = 0.01; HR = 2.8, *p* = 0.002; HR = 2.4, *p* = 0.005, respectively). When combined, the integrated biomarker worked as a much more accurate prognostic classifier for stage I adenocarcinoma (HR = 10.2, *p* = 3 × 10^−5^).

Importantly, abundant undegradaded microRNAs circulate in body fluids, such as blood, plasma, serum, and sputum [8]. There have been several studies to predict survival in NSCLC patients, by using microRNA profiles in body fluids. In 2010, Hu *et al.* reported that a four-serum-microRNA signature (*miR-486*, *miR-30d*, *miR-1*, and *miR-499*) was associated with overall survival in NSCLC, utilizing 120 cases for the training set and 123 cases for the testing set [52]. Subsequently, Wang *et al.* conducted pathway-based serum microRNA profiling and examined the prognostic association in advanced stage NSCLC patients [53]. They focused on microRNAs involved in TGF-β pathway, which plays crucial roles in control of cell proliferation, differentiation, apoptosis, and invasion. They identified 17 microRNAs which were significantly associated with two-year patient survival. Of these 17 microRNAs, *miR-16* exhibited the most statistically significant association; high expression of *miR-16* was associated with better survival (HR = 0.4, 95% confidence interval (CI), 0.3–0.5). They created 17-microRNA risk score to identify patients at the highest risk of death. NSCLC patients with a high-risk score had a 2.5-fold increased risk of death compared with those with a low-risk score (95% CI, 1.8–3.4; *p* = 1.1 × 10^−7^).

### 6.3. Let-7, DICER1, and Survival of Lung Cancer

DICER1 has an important role in microRNA biogenesis, converting precursor-microRNAs into mature microRNAs (Figure 1). Inversely, microRNA *let-7* directly down-regulates *DICER1* expression [20]. According to the study by Karube *et al.* [54], the reduced expression of *DICER1* was associated with poor prognosis in NSCLC, suggesting the involvement of reduced *DICER1* expression in the progression of lung cancer.

## 7. MicroRNA Associated with Driver Mutations and Therapeutic microRNAs

Somatic mutations in tyrosine kinases have recently emerged as driver mutations in carcinogenesis of lung cancer, especially in that of lung adenocarcinoma. Adenocarcinomas with activating mutations of *EGFR* are responsive to EGFR tyrosine kinase inhibitors (TKIs). However, T790M *EGFR* mutation and *MET* amplification lead to the resistance of EGFR TKIs [55]. Other mutually exclusive genetic alterations, which have been reported to work as driver mutations in lung adenocarcinoma, include translocations of *ALK*, *ROS1*, *RET*, *NTKR1*, *NRG2*, *ERBB*4, and *BRAF*, and mutations of *KRAS*, *BRAF*, *ERBB2*, *NRAS*, *HRAS*, *MAP2K1*, *NF1*, and *RIT1* [56,57,58,59,60,61]. Adenocarcinomas with specific driver mutations sometimes have characteristic clinicopathological features [62,63,64,65,66,67]. For example, *ALK*-translocation lung adenocarcinomas are characterized by young-onset, never/light smokers, and acinar morphology with mucin/signet-ring cell morphology [64,65,66,67].

There are several studies examining the association between microRNAs expression and driver mutations (especially *EGFR* and *KRAS* mutation). *miR-21* has been suggested to be an EGFR-regulated anti-apoptotic factor in never-smokers’ lung adenocarcinoma [68]. Recently, Li *et al.* have reported an association of *miR-21* overexpression with acquired resistance of EGFR-TKI in NSCLCs [69]. Dacic *et al.* [70] examined the microRNA expressions in lung adenocarcinomas with different driver mutations, focusing on *EGFR* and *KRAS* mutation, using two *EGFR*-mutant, two *KRAS*-mutant, and two *EGFR*-wild-type/*KRAS*-wild-type adenocarcinomas and confirmed differentially expressed microRNAs in a validation set of 18 adenocarcinomas. They showed that *miR-155*, *miR-25*, and *miR-495* were up-regulated only in the *EGFR*-wild-type/*KRAS*-wild-type, *EGFR*-mutant, and *KRAS*-mutant lung adenocarcinomas, respectively. In 2014, Bjaanaes *et al.* also examined microRNA profiles according to mutation status of *EGFR* and *KRAS*, using 154 lung adenocarcinomas by microarray [71]. The identified profiles were confirmed by real-time RT-PCR, using 103 lung cancer cases. They identified 17 microRNAs that were differentially expressed between *EGFR*-mutant and *EGFR*-wild-type adenocarcinomas, and three microRNAs differentially expressed between *KRAS*-mutant and *KRAS*-wild-type adenocarcinomas. There was no overlap between Dacic’s and Bjaanaes’ study, possibly due to the small sample size of Dacic’s study, differences in methodologies, or by chance. Further researches with a large sample size are required to elucidate the specific microRNA profiles according to the mutation status of *EGFR* or *KRAS*.

Weiss *et al.* [72] reported that *miR-128b* could directly regulate *EGFR*. The loss of heterozygosity (LOH) of *miR-128b* occurred frequently in NSCLC and correlated significantly with clinical response and survival after EGFR-TKI. Another study showed that *microRNA-146a* targeted *EGFR*, suppressed its downstream signaling and regulation of cell growth, and enhanced the cytotoxic effect of EGFR-TKI in five NSCLC cell lines [73].

To understand the role of microRNAs in TKI-resistant NSCLCs, Garofalo *et al.* examined microRNA changes that were mediated by tyrosine kinase receptors [74]. They demonstrated that this resistance could be overcome by anti-*miR-221/222* and anti-*miR-30c*, which recovered the expression of the pro-apoptotic protein BCL2L11 (BIM) and increased the gefitinib sensitivity of NSCLC both *in vitro* and *in vivo.*

Other aspects regarding the role of microRNAs in relation to EGFR-TKI deserve comment. Recently, plasma microRNA profiles (*miR-21*, *miR-27a*, and *miR-218*) have been identified for primary resistance to EGFR-TKIs in advanced NSCLCs with *EGFR* activating mutation [75]. Pak *et al.* have found unique microRNAs (*miR-34c*, *miR-183*, and *miR-210*) in lung adenocarcinoma groups according to major TKI sensitive *EGFR* mutation status [76]. A recent study by Wang *et al.* suggests that the modulation of specific microRNAs (*miR-374a* and *miR-548b*) may provide a therapeutic target to treat or reverse gefitinib resistance in NSCLC with high expression of the Axl kinase [77]. The review by Ricciuti *et al.* [78] is worth reading because it summarized the existing relationship between microRNAs and resistance to EGFR-TKIs, and also focusing on the possible clinical applications of microRNAs in reverting and overcoming such resistance.

For now, limited evidence can be available for the microRNA profiles of lung cancer with specific driver mutations. Further studies should be conducted to elucidate driver-mutation-specific microRNA profiles. MicroRNA signature may be used as a diagnostic biomarker of lung cancer with a specific driver mutation, and also the combination of TKI and microRNA-based treatment is promising.

## 8. Prospects from Basic to Clinical Application of microRNAs

Actually, despite enormous efforts, several obstacles remain to be solved for the transition of microRNAs from basic to clinical application as novel biomarkers or potential tools for treatment. These obstacles include the standardization of microRNA detection, improved understanding of how microRNAs interact with other components of the genome, and the development of non-toxic targeted delivery of microRNAs to the lung or metastatic lesions as well as the selection of the proper vehicle for delivery [79]. Therefore, we have a bunch of obstacles to clear away, however we are now trying to solve these issues strenuously [79].

## 9. Conclusions

In this review, we introduced recent works of microRNAs involved in the development and progression of lung cancer, with a particular interest in microRNAs as novel biomarkers and potential tools for treatment. Of importance, microRNAs are far less degradated in FFPE samples than mRNAs, and therefore, an easy availability of FFPE samples in hospitals enables us to measure accurate microRNA expressions. In addition, microRNAs are also present in body fluids, making microRNAs promising diagnostic biomarkers. Therefore, there exists a great potential in microRNA analyses for cancer research. Further studies are demanded in order to use microRNA profiles as diagnostic markers and conduct microRNA-based therapies in clinical practice.

## Figures and Tables

**Table 1:** Principal microRNAs involved in the development or progression of lung cancer.

microRNAs	Gene Targets	Biological Processes
Tumor suppressor microRNAs with down-regulation in lung cancer		
*let-7 family*	*RAS*, *HMGA2*, *CDK6*, *MYC*, *DICER1*	(i) Cell proliferation (*RAS*, *MYC*, *HMGA2*)
		(ii) Cell cycle regulation (*CDK6*)
		(iii) microRNA maturation (*DICER1*)
*miR-34 family*	*MET*, *BCL2*, *PDGFRA*, *PDGFRB*	TRAIL-induced cell death and cell proliferation
*miR-200 family*	*ZEB1*, *ZEB2*, *E-cadherin* (*CDH1*), *vimentin* (*VIM*)	Promotion of EMT and metastasis
Oncogenic microRNAs with up-regulation in lung cancer		
*miR-21*	*PTEN*, *PDCD4*, *TPM1*	Apoptosis, cell proliferation, and migration
*miR-17-92 cluster*	*E2F1*, *PTEN*, *HIF1A*	Cell proliferation and carcinogenesis
*miR-221/222*	*PTEN*, *TIMP3*	Apoptosis and cell migration

TRAIL: TNF-related apoptosis-inducing ligand; EMT: epithelial-mesenchymal transition.
